# Social and Biological Transgenerational Underpinnings of Adolescent Pregnancy

**DOI:** 10.3390/ijerph182212152

**Published:** 2021-11-19

**Authors:** Amanda Rowlands, Emma C. Juergensen, Ana Paula Prescivalli, Katrina G. Salvante, Pablo A. Nepomnaschy

**Affiliations:** Maternal and Child Health Laboratory and Crawford Laboratory of Evolutionary Studies, Faculty of Health Sciences, Simon Fraser University, Burnaby, BC V5A 1S6, Canada; emma_juergensen@sfu.ca (E.C.J.); apcosta@sfu.ca (A.P.P.); kgsalvan@sfu.ca (K.G.S.)

**Keywords:** adolescent pregnancy, Eco-social Theory (EST), Life History Theory (LHT), Developmental Origins of Health and Disease (DOHaD), inequities, interventions, menarche, coitarche, age at first birth, transgenerational effects

## Abstract

Adolescent pregnancy (occurring < age 20) is considered a public health problem that creates and perpetuates inequities, affecting not only women, but societies as a whole globally. The efficacy of current approaches to reduce its prevalence is limited. Most existing interventions focus on outcomes without identifying or addressing upstream social and biological causes. Current rhetoric revolves around the need to change girls’ individual behaviours during adolescence and puberty. Yet, emerging evidence suggests risk for adolescent pregnancy may be influenced by exposures taking place much earlier during development, starting as early as gametogenesis. Furthermore, pregnancy risks are determined by complex interactions between socio-structural and ecological factors including housing and food security, family structure, and gender-based power dynamics. To explore these interactions, we merge three complimentary theoretical frameworks: “Eco-Social”, “Life History” and “Developmental Origins of Health and Disease”. We use our new lens to discuss social and biological determinants of two key developmental milestones associated with age at first birth: age at girls’ first menstrual bleed (menarche) and age at first sexual intercourse (coitarche). Our review of the literature suggests that promoting stable and safe environments starting at conception (including improving economic and social equity, in addition to gender-based power dynamics) is paramount to effectively curbing adolescent pregnancy rates. Adolescent pregnancy exacerbates and perpetuates social inequities within and across generations. As such, reducing it should be considered a key priority for public health and social change agenda.

## 1. Introduction

Approximately 13 million adolescent girls (age < 20) give birth each year worldwide [1]. Adolescent pregnancies are linked to increased morbidity risks during gestation, birth and early post-partum, and are the leading cause of death among girls aged 12–19 years old globally [2,3,4,5,6]. Furthermore, adolescent pregnancies lead to a broad range of health sequelae for women. For example, compared to women who give birth between ages 20 to 24, adolescent mothers experience higher risks of pre-eclampsia and eclampsia [7], anaemia [8], puerperal endometriosis, and chronic infection [1]. Risk of abortion is also increased, with 5.6 million girls aged 15–19 undergoing the procedure annually [2]. Almost 70% of those abortions are practiced in unsafe conditions, risking girls’ lives, health and wellbeing [9]. Adolescent pregnancies also carry increased risks for negative birth outcomes including higher risk of stillbirth and preterm birth, in addition to low birth weight [7,8,10,11]. Children born to adolescent mothers also face higher risks of experiencing negative health and developmental outcomes [11]. Importantly, daughters of adolescent mothers are also at higher risk of becoming adolescent mothers themselves compared to those of older mothers [12,13,14]. This results in the transgenerational transmission of the risks associated with adolescent pregnancy, including increased risks of social isolation, depression, domestic violence and poverty [11]. In other words, adolescent pregnancy perpetuates inequities suffered by mothers and their lasting physical, social and mental health implications for their offspring. Consequently, adolescent pregnancy not only represents a global public health concern but also a social and gender-equity one.

### Limitations of Current Interventions

The extensive individual and social costs of adolescent pregnancy have motivated the development of multiple interventions aimed at decreasing its prevalence and combating its negative effects (Table 1). As reflected in Table 1, most existing programs are focused on modifying individual behaviours (“behavioural interventions”). These interventions include sexual education [15,16]; promoting behavioural strategies to delay sexual initiation and encourage abstinence [17,18,19,20]; and increasing contraceptive knowledge, access, and use [19,21,22]. Some of these programs, however, have been shown to have low efficacy. For example, a study conducted in 57 Australian high schools reported that girls enrolled in abstinence programs faced twice the risk of experiencing a pregnancy and an abortion before age 20 compared to those in the control group (8% vs. 4%, respectively) [20]. Some of these interventions do succeed at reducing adolescent sexual activity, increasing contraceptive use and decreasing adolescent pregnancy in some regions of the world (e.g., Canada [23]; the United States [24]; some European countries [16,25,26,27]). Yet, interventions solely aimed at changing individuals’ behaviours present important limitations because adolescent pregnancy is a complex outcome. Indeed, adolescent pregnancy is a consequence of the intersection of societal structures that generate risky environments for adolescent girls. Environments characterised by socio-structural and ecological inequities, including poverty; limited education; low employment; food, water, and housing insecurity; gender-based violence; and sexual coercion are associated with higher rates of adolescent pregnancy [1,2]. Therefore, to more effectively decrease the prevalence of adolescent pregnancy, interventions should target these upstream causes.

Importantly, although adolescent pregnancy is generally used to refer to girls under twenty years of age as a homogeneous group, its associated risks vary with age. For example, as girls age, their risk of facing cephalopelvic disproportion, which occurs when the foetus’ head cannot pass through the birth canal, decreases [28,29]. This is important because cephalopelvic disproportion and other complications during delivery rank amongst the top causes of maternal mortality [30], in addition to being associated with increased risks for several other negative birth outcomes [29]. Furthermore, while social norms regarding coitarche and marriage or other forms of reproductive partnerships for adolescents vary across socio-structural and ecological contexts, the stigma and other social costs associated with adolescent pregnancy tend to lessen with age. Access to social or economic resources, by comparison, tend to increase with age within adolescence across different socio-structural and ecological contexts. Thus, the net cost associated with pregnancy tends to decrease with age during adolescence. Yet, adolescent pregnancy studies rarely consider either chronologic or gynaecologic (time since menarche) age-related variation in outcomes, often lumping all girls under 20 years of age into one category and comparing them as such against adult women [5,31,32]. This approach is problematic as it can lead to the underrepresentation or overrepresentation of the risks faced by younger or older adolescent girls, respectively, in different contexts [32]. Thus, adolescent pregnancy interventions need to be sensitive to variation in maternal age among adolescents, in addition to the diverse socio-structural and ecologic contexts in which they are immersed.

**Table 1:** Examples of current interventions aimed at curbing adolescent pregnancy.

Intervention	Reference	Outcome	Developmental Period	Population	Socio- Economic Context	Level of Action	Location
School-Based Abstinence Only Sexual Education & School-Based Abstinence Plus Contraceptive Sexual Education (Review)	(Bennett & Assefi, 2005) [19]	**Not effective** Modest, short acting change to adolescents’ sexual behaviour; Programs with contraceptive information improved adolescents’ knowledge about contraceptives but did not significantly change their sexual behaviours	Adolescence	Girls and boys	Industrialised, variable SES	Individuals	USA
Primary Prevention Sexual Education Programs Delivered at Schools, Community Centers, and Health Clinics	(DiCenso et al., 2002) [15]	**Not effective** Does not significantly delay initiation of sexual activity, increase contraceptive use, or prevent pregnancies	Adolescence	Girls and boys	Industrialised (trend towards low-SES)	Individuals	Canada, USA, UK, Australia, New Zealand, Europe
School-Based Infant Simulator Program (students tasked with caring for a robotic infant)	(Brinkman et al., 2016) [20]	**Not effective** Compared to standard sexual education program, participants had a higher pregnancy risk, were more likely to experience birth and an induced abortion before age 20.	Adolescence (13–15 years)	Girls	Industrialised	Individuals	Australia
School-Based Infant Simulator Program (students tasked with caring for a robotic infant)	(Herrman et al., 2011) [33]	**Not effective** No significant changes in adolescents’ perception of pregnancy and parenting	Adolescence (14–18 years)	Girls and boys	Industrialised	Individuals	USA
Teens and Toddlers Intervention Program (at-risk adolescent girls enrolled in volunteer service in preschools to experience the reality of caring for children)	(Bonell et al., 2013) [22]	**Not effective** Did not significantly reduce rate of sexual activity without contraception or expectation of adolescent parenthood score	Adolescence (13–14 years)	Girls	Industrialised	Individuals	UK
School-Based Peer-Led Sexual Education (standard curriculum delivered by 16–17-year-old peers)	(Stephenson et al., 2004) [16]	**Positive partial results** Girls reported significantly lower intercourse frequency by age 16 compared to control group (teacher-led sexual education); No difference in boys’ behaviour; No difference in self-reports of adolescent pregnancy compared to controls at follow-up (18 months post-intervention)	Adolescence (13–14 years)	Girls and boys	Industrialised	Individuals	UK
School-Based Pregnancy Prevention Program using the I-Change Model (focused on attitude and behavioural change, teaching communication and negotiation strategies)	(Taylor et al., 2014) [18]	**Positive partial results** Increased report of condom use, plans to communicate with partners about pregnancy, and intentions to abstain from sex while at school; Of those who reported having had sex, no difference in self-reports of adolescent pregnancy compared to controls at follow-up (8 months post-intervention)	Adolescence (13–14 years)	Girls and boys	Developing	Individuals	South Africa
Expanded Access to Long-Acting Reversible Contraceptives (LARCs) (provision of free LARCs, increased promotion and education for at-risk individuals)	(Lindo & Packham, 2017) [21]	**Promising** Reduction in adolescent pregnancies; Authors suggest this result to be partially attributable to increases in LARC uptake	Adolescence	Girls	Industrialised	Structural	USA
Implementation Intention Setting (at-risk adolescent girls coached in contraceptive use intention-setting and communication of these intentions)	(Martin et al., 2009) [24]	**Promising** Significant shift from participants consulting family planning clinic for emergency contraception and pregnancy testing to consulting for contraceptive supplies only; Fewer positive pregnancy tests compared to controls at follow-up (9 months post-intervention)	Adolescence (mean 16.7 years)	Girls	Industrialised	Individuals	USA
Parent-Based Interventions (sexual education programs including parental involvement in educating about sexual health decision-making and behaviours)	(Widman et al., 2019) [17]	**Postive partial results** Improved condom use and parent–child sexual communication; No impact on delaying onset of adolescent sexual activity	Adolescence (younger than 18 years)	Girls and boys	Industrialised	Family Unit	USA
Mother–Daughter Communication Focused Intervention (multi-week sexual health education program featuring facilitated conversation between mothers and daughters)	(Powwattana et al., 2018) [34]	**Promising** Significant increase in frequency of discussions about sexual risk between mothers and daughters; Significant increase in perceived power in relationship control and ability to prevent sexual risk for daughters	Adolescence (12–15 years)	Girls	Developing	Family Unit	Thailand
Early Childhood Intervention and Youth Development Programs (designed to promote school engagement and raise life aspirations via career development and work experience for youth with adverse childhoods)	(Harden et al., 2009) [25]	**Promising** Lower adolescent pregnancy rates among individuals who received the opportunity to participate in an intervention program	Childhood Adolescence	Girls and boys	Industrial	Individuals	UK
Conditional Cash Transfers for Education (significant sums of money provided to adolescent girls contingent on their enrolment in and completion of education programs)	(Cortés et al., 2016) [35]	**Promising** Reduced adolescent pregnancy rates	Adolescence	Girls	Developing	Structural	Colombia

To achieve this aim it is necessary to shift the current framing of this public health issue so that we can move from interventions and policies focused on the individual to addressing the social and environmental structures that are at the root cause of adolescent pregnancy. Part of this shift involves changing the way that we ask questions about adolescent pregnancy and interpret the results obtained. This is a necessary step to avoid perpetuating inequities. Here we briefly review three complementary frameworks that we merge to achieve our aim: Eco-Social, Life History, and Developmental Origins of Health and Disease theories.

## 2. Combining Theoretical Frameworks to Tackle Adolescent Pregnancy

### 2.1. Eco-Social Theory

Eco-Social Theory (EST) provides a conceptual framework to evaluate the relationships among health inequities and power structures, and their transgenerational effects [36,37]. According to this theory, individuals live within socially ascribed categories and identities, which constitute intersecting social structures. These social structures perpetuate inequitable health and social outcomes via social narratives that sustain those inequities, and through systems such as housing, education, employment, income, benefits, law, property, health care, and social and criminal justice systems [37]. Therefore, how a public health issue, such as adolescent pregnancy, is understood and conceptualised affects the choice of tools used to address it. An eco-social lens can, thus, be useful to develop public health interventions that promote a shift to systems-focused interventions that target the underlying social structures. Doing so would help research and policy move away from “blaming the victim” approaches, which are conspicuous when it comes to women’s reproductive health. Inequitable social structures also promote environments that increase reproductive risks for women. Importantly, those social structures lead to power inequities that hinder the ability to enact risk avoidance strategies [38]. The current rhetoric used to sustain strategies aimed at curbing adolescent pregnancy via promoting individual behavioural changes often ignores the fact that risk avoidance is not a viable option for everyone and that such strategies are, thus, harmful.

The EST approach, reducing socio-structural and ecological risk factors, has been successfully applied to tackling other reproductive health issues; for example, the risk of contracting HIV increases with substance use, needle sharing, and sex work [39]. Individual-focused interventions aimed at changing said behaviours has had a limited effect on reducing risky behaviours. Thus, Aidala and colleagues (2005) decided to take an EST approach and evaluated the social and ecological contexts contributing to injection drug use behaviours. They found that housing insecurity, for example, was associated with a 2–4 fold increased risk of these behaviours [39]. Armed with this knowledge, they worked on improving housing stability and within nine months, observed the aforementioned risky behaviours fall by half [39]. These results are consistent with EST’s proposition that targeting upstream, socio-structural and ecological causes can, indeed, improve the outcomes of interventions.

We propose that this framework can also be applied to curtail adolescent pregnancy by targeting its upstream causes. Specifically, efforts should be aimed at gender-based power imbalances, gendered violence, and socio-economic disparities affecting women and girls [40,41,42]. Next, a critical question needs to be addressed: at what point during a woman’s life do these socio-structural and ecological factors begin to exert their effects? Most research on adolescent pregnancy has focused on exposures taking place around puberty and adolescence. We argue that events occurring much earlier in development may also play a critical role. Next, we justify this proposition by reviewing the determinants of two key factors associated with the risk of adolescent pregnancy: age at menarche and age at coitarche, which affect age at first birth. To do so we introduce principles from two other complementary theoretical frameworks: Life History Theory (LHT) and Developmental Origins of Health and Disease (DOHaD).

### 2.2. Eco-Social Theory Meets Life History Theory and Developmental Origins of Health and Disease

LHT investigates the evolution and ecology of life history strategies. According to its main principle, resources used for one metabolic task become unavailable for other tasks [43]. Within this framework, the variation observed in the pace at which girls develop, the onset of their reproductive maturation (menarche), and their subsequent fecundability (their chances of conceiving in any given ovarian cycle) can be understood as a result of trade-offs in metabolic energy allocation between reproductive development and somatic growth and maintenance. Consequently, the number and severity of socio-structural and ecological challenges and risks (the perception of future challenges) faced during development may affect the timing of menarche, coitarche and first birth. 

Importantly, the amount of metabolic energy invested in responding to challenges versus development does not only depend on energy availability but also on the predictability, frequency, and intensity of the challenges individuals face. For example, in low mortality environments where access to nutritional resources is limited, girls may be expected to experience slower paces of reproductive development. In this context, if safe, the optimal biological strategy would be to delay reproduction until somatic development is completed and accumulated metabolic energetic reserves are sufficient for successful reproduction [44]. In contrast, in risky environments with high mortality, such as areas where war or violence are frequent and unpredictable, girls may experience a faster pace of reproductive development, reducing their risk of dying without reproducing. This proposition is consistent with observations made in human and non-human species where risky environments have been associated with accelerated reproductive development [45,46,47,48,49,50,51].

DOHaD offers yet another complementary framework to investigate how social and ecological conditions may influence the risk of adolescent pregnancy. Starting at the onset of development, DOHaD takes a longitudinal approach to understanding health and disease [52,53]. Combined with EST and LHT, DOHaD can be used to investigate the effects of environmental exposures and the trade-offs they impose between somatic and reproductive development and their reproductive consequences. Additionally, DOHaD focuses on transgenerational effects, which promote the investigation of the role of environments prior to the moment an individual is conceived, at gametogenesis. The integration of these three complementary frameworks may, thus, further our understanding of the biological mechanisms through which social and ecological exposures may affect the risk of adolescent pregnancy.

## 3. Biological Mechanisms Linking Socio-Structural and Ecological Exposures to Reproductive Maturation

Emerging evidence supports the existence of links between socio-structural and ecological exposures and girls’ pace of reproductive maturation, and their ages at menarche and coitarche [8,54]. In turn, earlier age at both menarche and coitarche appear to be associated with earlier age at first birth [55,56]. Understanding the biological mechanisms mediating these links and identifying specific socio-structural and ecological risks and critical windows of susceptibility across development should inform socio-structural interventions aimed at reducing adolescent pregnancy risks.

### 3.1. Epigenetic Mechanisms

Epigenetic processes are one pathway through which environmental factors can influence physical, behavioural, cognitive and reproductive development [57,58,59,60,61,62,63]. Among those, DNA methylation and histone acetylation can influence gene transcription [58,64]. Although epigenetic modifications can occur throughout the lifespan [59,65,66], fertilisation and early embryogenesis appear to be important stages for epigenetic phenomena affecting ontogenetic trajectories of both girls and boys [67,68]. Early DNA methylation marks can be inherited within a cell lineage, amplifying their effects across related developing tissues and organs [64]. Therefore, even when some early methylation marks are lost during development, their early ontogenetic effects can significantly influence cell and tissue differentiation with a cascade of consequences for later development. Thus, DNA methylation modifications caused by early socio-structural and ecological exposures have the potential to modify the pace of reproductive maturation and reproductive outcomes [69,70,71,72,73,74].

Indeed, a number of studies have shown that DNA methylation can be involved in the regulation of menarche-related genes. For example, a study in humans found that DNA hypermethylation of several *ZNF* genes is associated with precocious puberty in girls [75]. Links have also been reported between increased DNA methylation and histone acetylation of the Polycomb group (PcG) of transcriptional silencers and the initiation of reproductive maturation in rats and non-human primates [76,77,78]. These epigenetic modifications alleviate PcG suppression of puberty-activating genes, including several menarche-related *ZNF* genes and *Kiss1*, which regulate the pulsatile secretion of gonadotropin-releasing hormone (GnRH)—an essential first step in reproductive maturation [76,77,78]. Importantly, although it is clear that epigenetic processes play a role in the timing of reproductive development milestones, it is not yet clear which socio-structural or ecological exposures trigger epigenetic modifications at each developmental stage.

### 3.2. A Role for the Stress Axis

A biological mechanism via which socio-structural and ecological exposures may affect development is through their effects on the ontogenetic trajectory (“programming”) of the hypothalamic–pituitary–adrenal (HPA) axis, or stress axis. The HPA axis is involved in the allocation of metabolic energy to meet energetic, immunologic or psychosocial challenges [79,80,81,82]. Thus, changes in HPA axis ontogeny can result in changes in energy allocation patterns during development and, consequently, in the endocrine and metabolic regulation of other biological systems, including the reproductive, or hypothalamic–pituitary–gonadal (HPG) axis. HPA axis programming begins before conception, during gametogenesis [63,83,84], and continues throughout gestation and early childhood [85,86]. Once functional, the HPA axis, in its energy regulation role, can affect the pace of growth and functional maturation of all other biological systems, including the HPG axis [87,88,89]. This occurs through the interaction of cortisol with glucocorticoid receptors located throughout the hypothalamus, pituitary and ovaries. These interactions regulate gonadotropin releasing hormone (GnRH), luteinising hormone (LH), follicle stimulating hormone (FSH), and oestrogen and progesterone synthesis and release [90]. It is through this HPA axis–HPG axis cross talk that early HPA axis programming has the potential to accelerate or delay girls’ age at menarche, coitarche and first birth [90].

## 4. Timing of Socio-Structural and Ecological Exposures

### 4.1. Pre-Conception

**Figure 1:**
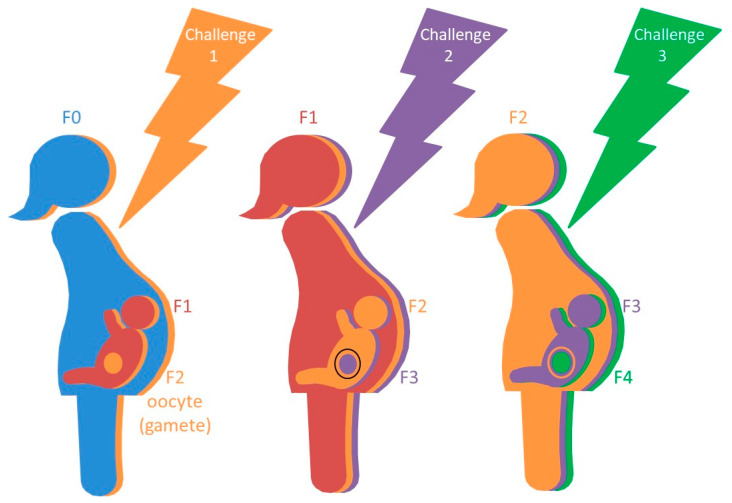
Transgenerational effects of prenatal exposures. Exposures faced by pregnant women (F0) can affect their unborn daughters’ (F1) gametes (F2), which begets the third generation (F2). These exposures may also have indirect effects on all subsequent generations (i.e., F3, F4, etc.). These effects can be mediated by epigenetic phenomena occurring when oocytes are undergoing their earliest developmental stages. The superposition of colours in the figure represents the direct and indirect effects that challenges in one generation may have on subsequent generations.

Socio-structural and ecological environments can start exerting their effects even before individuals are conceived, due to the effects that they exert on the germlines of grandparents and parents, which can be conserved transgenerationally. In mammals, oocyte ontogeny begins before birth. Women are born with all of their eggs and, thus, exposures faced by pregnant women (F0, “grandmother” generation) can affect their unborn daughters’ (F1, “mothers” generation) gametes, which begets the third generation (F2, “grandchildren” generation) (Figure 1) [63,83,91,92]. These exposures may also have indirect effects on all subsequent generations (i.e., F3, F4, etc.). These effects can be mediated by epigenetic phenomena occurring when oocytes are undergoing their earliest developmental stages (leptotene, zygotene and pachytene). Examples of germline epigenetic transmission have been reported for prenatal exposures such as stress [93,94], toxins and other chemical compounds [95,96], in addition to nutrient limitation and supplementation [97,98,99].

### 4.2. In Utero

After conception, the effects of socio-structural and ecological challenges on ontogenesis depend on the gestational stage at which each exposure takes place [100,101,102,103,104]. Epigenetic and ontogenetic schedules create critical periods of increased plasticity and vulnerability (“windows”) for particular tissues and systems. For example, the HPA axis starts to develop during the first few weeks post-conception with the differentiation of primordial cell lines that will give rise to the anterior pituitary, hypothalamus and adrenal cortex [105]. Consistent with this proposition, we have found evidence that suggests the existence of a link between early peri-conceptional maternal cortisol during the first 8 weeks post-conception and children’s HPA axis basal activity and stress responsivity at the onset of puberty [86]. Specifically, mothers’ cortisol during said period was associated with their 10- and 11-year-old daughters’ “basal” cortisol and HPA axis reactivity to natural (onset of the academic year) and experimental (the Trier Social Stress Test; [106,107,108]) challenges. In our search for potential mechanisms explaining these associations, we observed differential DNA methylation in 11 genes associated with maternal cortisol early post-conception, including genes in biological pathways involving corticotropin-releasing hormone (CRH) [86]. CRH is produced by the hypothalamus in response to stress. Yet, none of these DNA methylation profiles were associated with the daughters’ HPA axis basal activity or responsivity. Nonetheless, our results are consistent with those of other teams studying later gestational periods. Indeed, increased maternal anxiety and cortisol levels during the second and third trimesters of human pregnancy have been linked to elevated basal and stress-induced cortisol secretion and altered cortisol circadian profiles [109,110,111,112,113,114,115,116,117,118,119,120].

Consistent with the findings described above, major negative maternal life events during pregnancy recalled retrospectively have also been associated with altered HPA axis phenotypes of their offspring. Indeed, children of mothers exposed to intimate partner violence, divorce, death or severe illness of a close family member, marital infidelity, and serious financial problems have been reported to experience lower pre-stressor cortisol levels and increased adrenocorticotropic hormone and cortisol reactivity [84]. For example, in a study by Martinez-Torteya and colleagues (2016), intimate partner violence (IPV) perpetrated against mothers during gestation was linked with higher cortisol responses to experimental challenges in their 10-year-old children than that of children in the control group [121].

### 4.3. Post-Natal

Challenges and risk experienced during childhood also appear to affect the timing and pace of women’s reproductive maturation and age at first birth. LHT predicts that risk should affect the pace of reproductive maturation and the timing of first reproduction [45,46,47,48,49,50,51]. In high mortality contexts, adopting a “fast” life history strategy may be adaptive. This prediction is consistent with the results of several studies. Griskevicius and colleagues (2011), studying how mortality impacts reproductive timing, reported that violent crime rates, which are directly associated with high mortality rates, were associated with earlier age at first birth [45]. Similarly, Lynch and colleagues (2020) found that young Finnish girls who volunteered in paramilitary camps during World War II and were exposed to high mortality rates had earlier ages at first birth and shorter inter-birth intervals than their peers and sisters who did not volunteer [122]. As with the case of exposures taking place pre-conception or in utero, mechanistically, epigenetic processes may be at play.

In humans, high mortality risk environments, such as those characterised by frequent famines, natural disasters and other traumatic challenges, have been reported to be linked to modifications of DNA methylation profiles [123,124,125,126,127,128]. Similarly, alterations to DNA methylation profiles have been linked to chronic, inequitable social structures, such as discrimination, racism, low SES, and their long-term associated risks. For example, in Black and Latina women in the United States, perceived racism and discrimination have been associated with differential DNA methylation of stress-regulatory and disease-related (schizophrenia, bipolar disorder, and asthma) genes [129,130]. Comparisons of children aged 9–11 from families who differ in SES, parental education and family adversity levels have shown differences in DNA methylation profiles of genes related to the regulation of skeletal growth and immune function [131]. Importantly, the impact of early environments can continue to be observed in adults’ DNA methylation patterns, suggesting that the embedding of socio-structural and ecological exposures can persist across the lifespan [132,133].

At the physiologic level, interactions between the HPA axis and HPG axis may provide the mechanistic explanation for the link between high-risk environments and earlier reproductive maturation. A longitudinal study conducted by Ruttle and colleagues (2015) on adolescents in the US reported that the HPA axis–HPG axis relationship changes as girls transition from childhood to adolescence [134]. In childhood, several HPA axis and HPG axis hormones, including cortisol, dehydroepiandrosterone (DHEA) and testosterone, have been shown to be positively correlated, whereas their profiles are negatively correlated during adolescence [134]. The timing of this switch may depend on the level of stress experienced by the individual. Higher risk environments trigger more HPA axis activation. Ruttle and colleagues go on to show that girls who faced early life stress showed an earlier switch to a negative correlation between HPA axis and HPG axis hormones compared to girls who were not exposed to early life stress (at age 13 vs. age 15 in controls) [134]. These findings are consistent with the hypothesis that challenges and perceived risks in early life can lead to acceleration of reproductive maturation.

### 4.4. Family Dynamics

Family structure and dynamics may also affect the pace of reproductive maturation. Indeed, challenging familial exposures, including parental separation [135,136], having an adolescent mother [12,14,54,137], lack of parental support [44,138,139], parental control [140], low socioeconomic status [138], physical or sexual abuse, and parental substance use [141], and other family stressors [142], have all been reported to accelerate reproductive development in girls, including earlier ages at menarche, coitarche and first birth. In contrast, stable, supportive parenting environments have been associated with slower reproductive maturation, including later menarche and development of secondary sexual traits, and later coitarche [143,144].

### 4.5. Social Dynamics

Social dynamics, such as gender-based inequities, can also affect the pace of reproductive maturation and age at first birth. One critical form of gender-based inequity is sexual coercion. Women are significantly more likely to report being coerced into sex compared to men [145,146]. Importantly, the risk of experiencing sexual coercion is higher for girls who begin reproductive development or sexual activity at an earlier age [147,148,149]. Coercion may include guilt, lies, and direct, indirect, or perceived threats (such as the possibility of sexual violence or losing access to food or shelter). The likelihood of sexual coercion varies within and between communities, as it depends on the level of gender inequities and the distribution of resources. This risk may be accentuated in contexts of housing instability, food insecurity, or other unsafe conditions, as adolescents’ access to these social and material resources may be contingent on their engagement in sexual activity [150,151]. Indeed, resource scarcity during childhood has been found to influence the relationship between mortality cues and attitudes towards earlier reproduction. Consistent with this proposition, Griskevicius et al. (2011) found that individuals who reported feeling poor during childhood had a desire to have their first child and get married sooner, while those who reported feeling wealthy during childhood expressed a desire to delay reproduction and marriage [45]. Yet, it is important to note that sexual coercion can and does occur across all SES. This risk is not only determined by gendered power structures and how those structures may limit women’s abilities to defend themselves or their access to materials and social resources, but also by women’s perception of which of those resources are important or indispensable. Critically, that perception is not independent of the gender roles and expectations with which women are socialised in every culture and SES group [152].

## 5. Discussion and Conclusions

### 5.1. Targeting the Root Causes of Socio-Structural Inequities

Current rhetoric placing the responsibility of avoiding pregnancy on adolescent girls needs to be challenged and changed. This discourse is at the base of interventions that often revolve around rational decision making by individuals. Not only does this rhetoric blame and shame individual girls for “their behaviour”, but also ignores the constraints that socio-structural factors impose on biological development and its effects on reproductive outcomes. Prevalent discourses tend to assume that adolescent girls always have the ability to avoid risks. These assumptions fail to consider power asymmetries associated with age, gender and socioeconomic level. They also fail to consider the effects that socio-structural, ecological and physical environmental factors can have on the pace of reproductive maturation, coitarche and age at first birth. Our evaluation of these factors suggests that to reduce the risk of adolescent pregnancies, it is paramount to ensure that girls and women have access to critical resources throughout their lifespan.

### 5.2. Targeting Earlier Stages of Development

The framework emerging from combining EST, LHT and DOHaD may help us change current paradigms—how we think about, intervene on, and approach adolescent pregnancy and its subsequent risks to health and wellbeing. EST proposes that, to reduce the risk of adolescent pregnancy, it is critical to understand and address its upstream structural causes. Addressing upstream inequities would be more effective than attempting to change individuals’ behaviours that these inequities appear to promote. According to LHT, it is risky environments that lead to faster life histories, including both biological and behavioural strategies. Most existing interventions target girls who are already going through menarche. Yet, the onset and pace of reproductive maturation appear to be affected by exposures taking place much earlier, starting even before conception. Indeed, challenges faced by parents and grandparents may be passed on transgenerationally through their effects on germlines. DOHaD research results appear to be consistent with these principles as interventions targeting earlier stages of development tend to be more effective than those that start in adolescence.

### 5.3. Addressing Adolescent Pregnancy Involves Everyone

As discussed, an important shortcoming of most existing interventions for adolescent pregnancy is that they place the burden and responsibility on girls. Indeed, adolescent girls report greater perceived responsibility and face higher levels of social stigma compared to their male partners for early sexual behaviours and their outcomes, including not only adolescent pregnancy, but also the spread of sexually transmitted infections [153,154]. However, although boys and men are also critical participants in these behaviours, they are seldom included in related interventions. The societal norms that underly this gender-based disparity perpetuate the “victim blaming” model in response to related problems including sexual coercion and rape [155,156,157,158].

For interventions to be effective, we need positive, strength-based approaches that involve all genders and all ages. Having early and supportive sex-positive communication with parents and mentors that are appropriate for each life stage is paramount. Interventions need to foster the development of healthy relationships and emotional intelligence and facilitate learning about consent and conflict resolution. To increase girls’ and women’s sexual autonomy and dismantle the culture of sexual coercion and rape, programs are needed that ensure all members of society learn about power differentials. This is critically important as these power differentials limit individual decision-making processes. Importantly, individual perceptions regarding gender inequities can be hidden by privilege, particularly for those atop the privilege pyramid. It will only be through approaches that involve everyone that we will be able to reduce adolescent pregnancy risks.

### 5.4. Socio-Structural and Ecological Variation in Attitudes towards Adolescent Pregnancy and Its Outcomes

The majority of studies on adolescent pregnancy have been conducted in socio-structural and ecological contexts where adolescent pregnancy is stigmatised and considered an undesired outcome that needs to be curbed. Studies conducted in contexts where adolescent pregnancies are either the norm or are welcomed are scarce [32]. Within the latter contexts, adolescent motherhood is not always stigmatised and is, in some cases, supported by extended kinship networks that can provide allomaternal care, and other forms of social, emotional, logistic and economic support [32,159]. In these contexts, the social risks and their associated costs faced by adolescent mothers may be mitigated. Indeed, some authors argue that, within these supportive contexts, pregnancy outcomes between adolescent and adult mothers would be similar [32,160,161]. The accuracy of these claims is still being discussed, particularly for the youngest of mothers—those who have not completed their own development. To advance this debate it is paramount that new studies investigate adolescent pregnancy across diverse socio-structural and ecological contexts using “situated” approaches. These studies should explore both within- and between-population comparisons of risks across the full range of adolescents’ biological, social and emotional developmental stages. Their results should aid in the development of more nuanced approaches in terms of the design of interventions aimed at curbing the negative health outcomes associated with adolescent pregnancy. 

### 5.5. Breaking the Transgenerational Cycle of Adolescent Pregnancy

Experiencing pregnancy before the completion of biological, emotional and social development can carry long-term negative effects for both mothers’ and their children’s health and wellbeing. Thus, adolescent pregnancy can seriously impact adolescent mothers and their children’s opportunities in life. In so doing, in many social contexts, adolescent pregnancy may exacerbate and perpetuate social inequities within and across generations. Indeed, the risk of adolescent pregnancy may begin to increase before menarche, perhaps before conception. It may even originate from exposures affecting mothers and grandmothers. Curbing systemic inequities including housing and food insecurity, gendered power dynamics, sexual coercion and violence, and responsibility imbalances is paramount to breaking the transgenerational inheritance of inequities. Thus, it is of utmost importance that socio-structural interventions aimed at reducing this risk should target matrilineages and involve all sectors of society.

## Data Availability

No new data were created or analyzed in this study. Data sharing is not applicable to this article.
